# Stretchable and Washable Strain Sensor Based on Cracking Structure for Human Motion Monitoring

**DOI:** 10.1038/s41598-018-31628-7

**Published:** 2018-09-05

**Authors:** Jarkko Tolvanen, Jari Hannu, Heli Jantunen

**Affiliations:** Microelectronics Research Unit, Faculty of Information Technology and Electrical Engineering, University of Oulu, Oulu, 90014 Finland

## Abstract

Stretchable and wearable strain sensors have been intensively studied in recent years for applications in human motion monitoring. However, achieving a high-performance strain sensor with high stretchability, ultra-sensitivity, and functionality, such as tunable sensing ranges and sensitivity to various stimuli, has not yet been reported, even though such sensors have great importance for the future applications of wearable electronics. Herein, a novel and versatile strain sensor based on a cracking (silver ink patterned silicone elastomer)-(silver plated nylon structure) (Ag-DS/CF) has been designed and fabricated. The unique structure combined precisely shaped stretchable conductive fabrics and wrinkled Ag-ink pattern to achieve an excellent electrical performance. The Ag-DS/CF could be used to detect both large and subtle human motions and activities, pressure changes, and physical vibrations by achieving high stretchability up to 75%, ultrahigh sensitivity (gauge factor >10^4^–10^6^), tunable sensing ranges (from 7 to 75%). Excellent durability was demonstrated for human motion monitoring with machine washability. The extremely versatile Ag-DS/CF showed outstanding potential for the future of wearable electronics in real-time monitoring of human health, sports performance, etc.

## Introduction

Stretchable electronics have become one of the most vigorously studied research fields as they enable close fitting on curvilinear surfaces or direct attachment to the human skin that is highly important in new generation of portable and wearable electronics^1,2^. One of the key applications of stretchable electronics have been electronic skins that can mimic its functions, or even beyond that by enabling conversion of various stimuli produced by the human body and surrounding environment to electrical signals (pressure, strain, temperature, humidity, etc.)^3,4^. These can be widely applied in biomedical sensing for personalized healthcare^1,3–9^ or sports performance monitoring^3,5,8,9^, and human-machine interfaces for robotics, etc^1,3–6,10^.

In recent years, several studies have reported highly flexible and stretchable micro- and nanostructured pressure and strain sensors based on polymers embedded with electrically active materials such as silver or gold nanowires (AgNW or AuNW)^6,7,11,12^, carbon nanotubes^9,13–21^ (CNT), graphene^22–26^, graphite^24–26^, and other materials including inherently conducting polymers^1,3–5,10,27–34^. Achieving large stretchability (ɛ > 50%) and a high sensitivity (GF > 100) with a wide detection range have been the key factors in the development of strain sensors. This has been proven to be difficult^9,20,22,34,35^ because large stretching requires structural integrity, whereas substantial changes in the electrically active area are required in order to achieve high sensitivity^36,37^. However, several studies have shown that this is possible with highly sensitive piezoresistive structures^11,13,16,20,34,38,39^, but these sensors often show high contact resistance further limiting their feasibility to wearable sensing. Additional functionalities by fabricating transparent^14,16,19,26,27^, self-healing^40^, self-powered^3,41^, tunable^13,17,19,42^, multidimensional^7,12^ and multisensing (strain, pressure, temperature, etc.)^3,4,6,10,21,29,32,33,43,44^ strain sensors have been investigated. However, in many cases these added functionalities have made large stretchability, high sensitivity with low contact resistance, and wide detection range difficult to achieve.

Herein, highly functional piezoresistive strain sensor based on cracking (Ag-ink patterned silicone)-(silver plated nylon) structure has been fabricated. The novel strain sensor utilized wrinkling of Ag-ink pattern to accommodate the applied strain through expansion of the wrinkles. The machine washable strain sensor provided low contact resistance, ultrahigh sensitivity (GF > 10^5^–10^6^), tunable sensing ranges (7 to 75%), high durability, and fast response time. The sensor enabled detection of various stimuli (strain, pressure, bending, and physical vibration). Their extraordinary functionality and sensing performance enabled detection of various human motions and activities ranging from large to subtle.

## Results and Discussion

**Figure 1:**
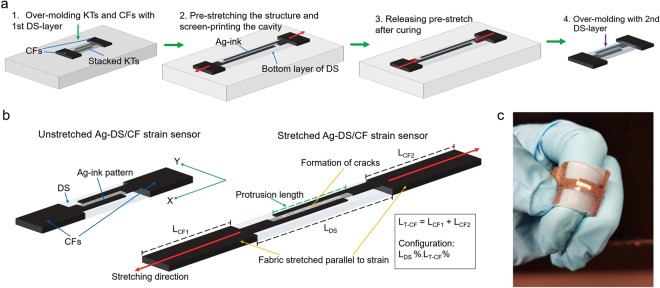
Fabrication, working mechanism, and photograph of Ag-DS/CF strain sensor. Fabrication processes of Ag-DS/CF strain sensors (**a**). The working mechanism of Ag-DS/CF strain sensor (**b**). The Ag-DS/CF form cracks perpendicular to direction of applied strain, whereas the fabric stretches parallel to the applied strain. The sensor configuration is expressed as [length of the over-molded part (L_DS_)]-[length of the CFs on both sides (L_CF1_ and L_CF2_) of the over-molded part] in their percentages for the total length of the sensor (L_T_ = L_DS_ + L_T-CF_). Photograph of the fabricated sample of Ag-DS/CF (**c**).

Figure 1a shows the fabrication processes for the (Ag-ink patterned silicone)-(silver plated nylon) (Ag-DS/CF) strain sensors. The sensor configuration was expressed as the length of the over-molded part (Dragon Skin; DS) (L_DS_) to the length of the T/L-shaped (see Fig. S1) conductive fabrics (CFs) on both sides (L_T-CF_ = L_CF1_ + L_CF2_) of the over-molded part in terms of their percentages for the total length of the sensor structure, thus as L_DS_ %:L_T-CF_ % (Fig. 1b). For example, L_total = _L_T-CF_ + L_DS_, so if L_T-CF_ = L_DS_ then the configuration is 50:50. The sensor configuration 50 (short form of 50:50) was chosen with protrusion contact area lengths of approximately 95% of L_DS_ before pre-stretching. Thus, the L_CF1_ + L_CF2_ was equal to that of L_DS_. This was followed with adjustment of the volume of the cavity between protrusions of CFs by changing the size and number of stacked Kapton tapes (KTs). Cavity dimensions of 5 mm × 1 mm × 0.2 mm were used.

The fabricated structure of the Ag-DS/CF was homogenously pre-stretched to approximately 150% before screen-printing the cavity with the Ag-ink. The pre-stretching was released after curing, causing buckling-induced wrinkling of the Ag-ink pattern. The method was used because a non-wrinkled pattern could not withstand the strains during dynamic loadings. This was because of the formation of large and irreversible cracks perpendicular to the applied strain (X-axis) due to poor adhesion of the ink to the silicone elastomer. The buckling mechanism (e.g. wrinkled or wavy structure)^9,15,37,45^ has been proven to be an effective strategy for fabricating stretchable devices from non-stretchable materials^36^, as the applied stress can be effectively absorbed by expansion of the buckled structure until fully straightened, and also the adhesion of the ink is improved^46^. Thus, the stretchability performance was dependent on the level of pre-strain^47^ and electrically active areas of Ag-DS/CF were observed to remain intact during repeatable stretching up to strains of 75% in the direction of the X-axis for configuration 50. Regardless, the fabrication process offers a simple, low-cost, and scalable solution with the possibility for it to be large-area compatible.

### Sensing mechanism in Ag-DS/CF

**Figure 2:**
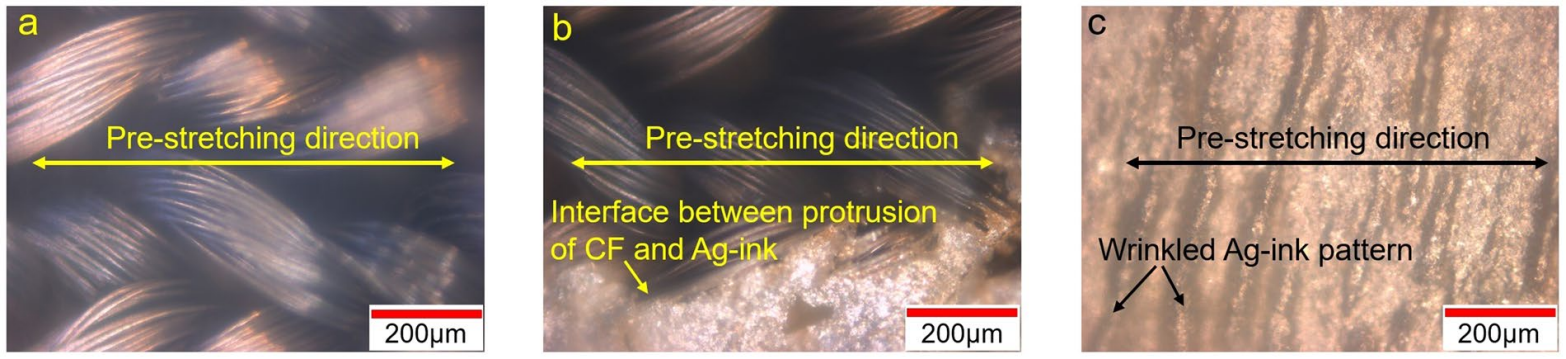
Optical microscopy images of Ag-DS/CF. The optical microscopy images of the knitted structure of CFs in Ag-DS/CF (**a**), interface between protrusion of CF and Ag-ink in Ag-DS/CF (**b**), and wrinkled Ag-ink pattern in pre-stretching direction in Ag-DS/CF (**c**), with scale bars of 200 µm and when unstretched.

Figure 1b presents the sensing mechanism of the Ag-DS/CF strain sensor shown in Fig. 1c during stretching. Different strain responses could be achieved depending on the sensor configuration employed in fabrication (e.g. L_DS_%:L_T-CF_%), and the attachment of the sensor during operation. The high protrusion percentage of 95% of CFs in relation to the Ag-ink pattern length significantly decreased the initial contact resistance to approximately 50–300 Ω with some variation between samples (Fig. S2). It also increased the stability and performance of the structure during dynamic loadings. When considering the behavior of Ag-DS/CF 50 (short form of 50:50) sensors at low strains, the CFs started to elongate (Fig. 2a,b) parallel to the applied strain (X-axis) and the wrinkled pattern slowly started to straighten (Fig. 2c). At this point, only small relative resistance changes could be seen, as the structural changes were mainly attributed to elongation of the CFs outside the over-molded DS structure. Further increase in the strain increased the total stretching of the CFs, whereas the straightened wrinkled Ag-ink pattern started to show small reversible fractures with the formation of small cracks perpendicular to the applied strain (Y-axis). At even higher strains, the CFs stretched further, and the average crack opening distance started noticeably to increase and disconnections between the CFs and Ag-ink pattern also increased (Fig. S3) leading to switch-like behavior at a certain strain level giving rise to significant resistance change. The microcracking mechanism has been widely applied in the fabrication of ultrasensitive strain sensors^20,27,35,48–50^. However, in these cases the stretching of these structures had to be severely limited, in some cases due to the unstable structure that would lead to irreversible electrical performance such as contact resistance increase or change in the response to an applied strain.

The strain response mechanism of sensor configuration 50 was greatly affected by the T/L-shaped CFs working as springs (Fig. 2a,b), absorbing the strain induced by stretching in the X-axis direction and distributing it over a larger area when the L_T-CF_ % was large. For other sensor configurations such as 100 (L_total_ = L_T-CF_ + L_DS_, so if L_T-CF_ = 0 and L_DS_ = 1 then the configuration is 100:0) the shape of the CFs changes from T/L to L (see Fig. S1). As a result, the applied strain significantly stretched the electrically active area of the Ag-DS/CF even more, as the absorption by the CFs and distribution of strain to a larger area in the structure decreased significantly due to the missing T-shape of the CFs. Also, the strain sensor could be stretched in the direction of the Y-axis, but this would change the sensing mechanism as the applied wrinkles created by pre-stretching and the stretchability of the knitted structure of the CFs is in the X-axis direction. At low strains, the protrusions of the CFs were observed to start dislocating from the Ag-ink pattern, leading to some increase of the contact resistance. When the strain increased, the wrinkled-pattern was easily disconnected by the formation of a large crack/cracks in the Y-axis direction. However, the strain sensing properties of the Ag-DS/CF in the Y and XY-direction require further study.

### Strain sensing of Ag-DS/CF

**Figure 3:**
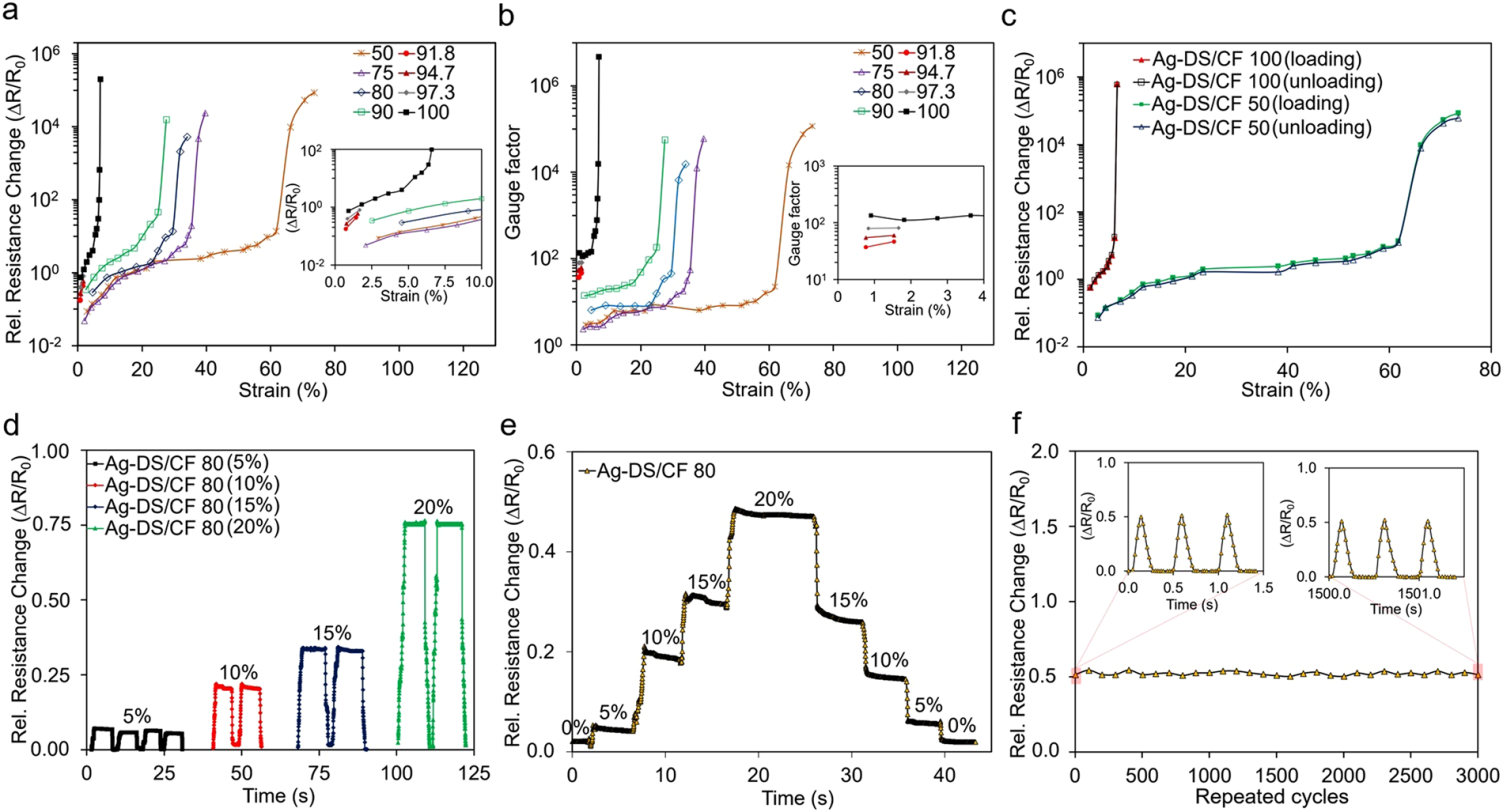
Measurement results for strain. Relative resistance change versus applied strain of Ag/DS-CF with different sensor configurations (L_DS_% − L_T-CF_%) (**a**). The GF versus applied strain of Ag-DS/CF (R_0_ = 263–300) (**b**). Recorded hysteresis curve of Ag-DS/CF during loading- unloading cycles (R_0_ = 263) (**c**). The time-dependent response of Ag-DS/CF 80 sensor configuration under dynamic loadings with strains of 5–20% (R_0_ = 263) (**d**). Step and hold test for Ag-DS/CF (R_0_ = 300) (**e**). The relative resistance change of Ag-DS/CF 80 versus applied strain of 20% for long-term stability test at 3000 repeated cycles (R_0_ = 300) (**f**).

Figure 3a shows the logarithmic relative resistance change (ΔR/R_0_) of Ag-DS/CF strain sensors versus applied strain. At low strain levels the relative resistance change increased linearly in all cases with increasing applied strain. A significant, exponential increase in the resistance to ΔR/R_0_ values of 10^4^–10^5%^ with tunable working ranges was observed at approximately 6, 25, 30, 35, and 62% strains for structures 100, 90, 80, 75, and 50 (short forms of 100:0, 90:10, etc.), respectively. The observed non-linearity of the strain response is typical for any type of piezoresistive strain sensor with a high GF. The Ag-DS/CF sensors demonstrated a reversible cracking mechanism for applied strains ≤75% (50% of the pre-strain level) but, depending on the sample, further stretching to ≥100%, was observed to lead to instantaneous irreversible resistance change and large-scale mechanical failure. The large resistance change demonstrated here is highly desirable for strain sensing applications, enabling high sensitivity to detect subtle changes of strain. Furthermore, the Ag-DS/CF displayed wide and highly tunable sensing ranges, where the relation between strain and resistance could be very finely tuned (e.g. 91.8, 94.7, etc.). The stress-strain curve of the sensor showed linearity at strain of <120% with low Young’s modulus of 0.0025 GPa (Fig. S4), whereas the slope increased when further strained. This is due to more limited stretchability of CF, and the stretching of the area covered by DS becomes more severe. The unique structure provides extremely versatile tuning possibilities in the strain-resistance relation during or after the fabrication process, e.g. by adjusting the pre-strain level, changing the value of L_DS_%:L_T-CF_%, or by adjusting the size of the CFs and/or the area of the DS layers to cover more of the internal structure. In this study, only the effect of adjusting the length-to-length ratio of CFs to Ag-ink pattern after fabrication of Ag-DS/CF was demonstrated.

The strain sensors’ sensitivity that defines the gauge factor is expressed as GF = (ΔR/R_0_)/(ΔL/L_0_). In Fig. 3b, depending on the value of L_DS_%:L_T-CF_% of the Ag-DS/CF, GFs between 36–140 and 2.3–19.7 were achieved at low strains of 0.7 to 1.7% and 2 to 10% with configurations of 100, 97.3, 94.7, and 91.8, and 90, 80, 75, and 50, respectively. When the applied strain was further increased, extremely high sensitivities up to >10^5^–10^6^ at strains of approximately 6–70% were achieved. These are among the highest reported GFs in strain sensors up to this date^11,19,20,25^. Furthermore, to the best of our knowledge, the Ag-DS/CF showed the highest achievable GF of 1.2 × 10^5^ at >70% strain that has ever been reported (Fig. S5). The ultrasensitivity of Ag-DS/CF was achievable because of excellent electrical contact between the CFs and Ag-ink pattern. This decreased the initial contact resistance to values of approx. 50–300 Ω. The cracking mechanisms of the Ag-ink pattern significantly increased the contact resistance to >10 MΩ when applying sufficient strain. The sensing mechanism in Ag-DS/CF is highly reversible and the extremely high GF is not associated with irreversible fracture leading to an infinite gauge factor.

Typically, high GF strain sensors show high hysteresis behavior that can impair the electrical performance during continuous dynamic loadings. Figure 3c shows a negligible hysteresis of ∼3% for Ag-DS/CF 100 whereas a significant hysteresis of ∼20% was observed for Ag-DS/CF 50, during dynamic loading-unloading cycles with strains of 0–6 and 0–75%, respectively. The sensor configurations 75, 80, and 90 showed hysteresis of approx. 5–13% with slightly decreasing value as a function of the length of the CF (Fig. S6). The low hysteresis observed in the 100 device indicated the improvement in the binding of Ag-ink pattern to the silicone elastomer. Also, the sliding of Ag-ink pattern past the cavity and inside the structure of Ag-DS/CF was effectively prevented, as both have been previously been suggested as sources of significant hysteresis in strain sensors^35^. The high hysteresis in Ag-DS/CF 50 could be related to different stretching-recovery behavior of the CFs as the T/L-shaped CFs (Fig. S1) acted as springs absorbing the strain induced by stretching and distributing it over a larger area when the L_T-CF_ increased. Thus, the strain at the center of the structure covered with DS varies and affects the electrical response of the Ag-DS/CF.

Figure 3d shows the dynamic performance when stretching-releasing cycles were applied to Ag-DS/CF at strains of 5–20% with a relatively constant frequency of approximately 0.10–0.14 Hz. The relative resistance changed proportionally with the applied strain, as expected, and was consistent with the ΔR/R_0_ curve in Fig. 3a when the sample variation was taken into account. Regardless of this, the dynamic performance of the Ag-DS/CF showed good stability and reproducibility at various strains. Furthermore, the Ag-DS/CF responses would not be expected to show frequency dependency at ≤2 Hz, frequencies important for human motion, as Fig. 3e–f show similar responses (ΔR/R_0_) when strain remains constant. The measurement precision and non-symmetrical response observed during loading was related to the inaccuracy of the used stretching tester that was not motorized and was not precisely controllable.

A step-and-hold test applied to observe the time-dependent resistance behavior of Ag-DS/CF related to overshooting and undershooting (Fig. 3e). The strain sensors were stretched and released by 5% within each step between 0–20% with a strain rate of approximately ±8% s^−1^ and the strains were held at each step for approximately 5 s. The Ag-DS/CF showed consistent and precise responses with low overshoots of ∼2.5% at strains of 0–20% (Table S1) indicating the reliability of the strain sensor. The overshooting was mainly related to the viscoelastic nature of the silicone elastomer, and would be expected to increase with higher strain and faster strain rate. Furthermore, the small hysteresis behavior (Fig. 3c) of Ag-DS/CF 100 seemed to be correlated to the time-dependent resistance relaxation, seen as a small overshoot in Ag-DS/CF 80 (Fig. 3d). Additionally, in Fig. 3c–e, the differences in ΔR/R_0_ for the Ag-DS/CF was the result of sample variation, mainly attributed due to variation of R_0_ between different samples (Fig. S2).

Figure 3f shows that Ag-DS/CF devices exhibited high stability during 3000 repeated loading-unloading cycles at a frequency of approximately 2 Hz with an applied strain of 20%. These resistance change curves were recorded after every 100 cycles. The high cyclic performance and excellent durability of the Ag-DS/CF is indicated by the negligible change in the response and no degradation of the GF, and is the result of the simple and effective structure preventing the various types of mechanical failures associated with sliding, delamination, or undesired cracking of the Ag-ink pattern during repeated stretching.

The first sign of mechanical failure in an Ag-DS/CF was seen after >10 hours of continuous testing with various strain levels at different locations on the human body after both durability testing for strain and pressure were completed. As a result, the initial resistance of the Ag-DS/CF increased significantly from approximately 300 to 2100 Ω. However, the GFs of the strain sensor did not decrease as seen in many other cases, but rather were observed to increase as the deformations in the Ag-ink pattern increased to the initial non-stretched stage, leading to a shift in the response towards the point of exponential increase in the relative resistance at smaller strains. After this type of failure, the Ag-DS/CF can still have a recoverable strain response, but response can be highly varied due to induced deformations and cracks causing unpredictable behavior upon applying strain.

However, the Ag-DS/CF structure remained intact and showed no degradation in electrical properties following up to 5 cycles of machine washing at a temperature of 40 °C, 1200 rpm, and 30 minutes per cycle (Fig. S7). Typically, the durability of strain sensors is only tested at strains correlating to 10–20% of the stretchability of the structure, with lower frequencies of ≤1.5 Hz, and the number of cycles between 1–10 k (Table S1). Thus, the Ag-DS/CF indicates a promising route to fabricate even more durable sensors than previously reported.

### Pressure sensing of Ag-DS/CF

**Figure 4:**
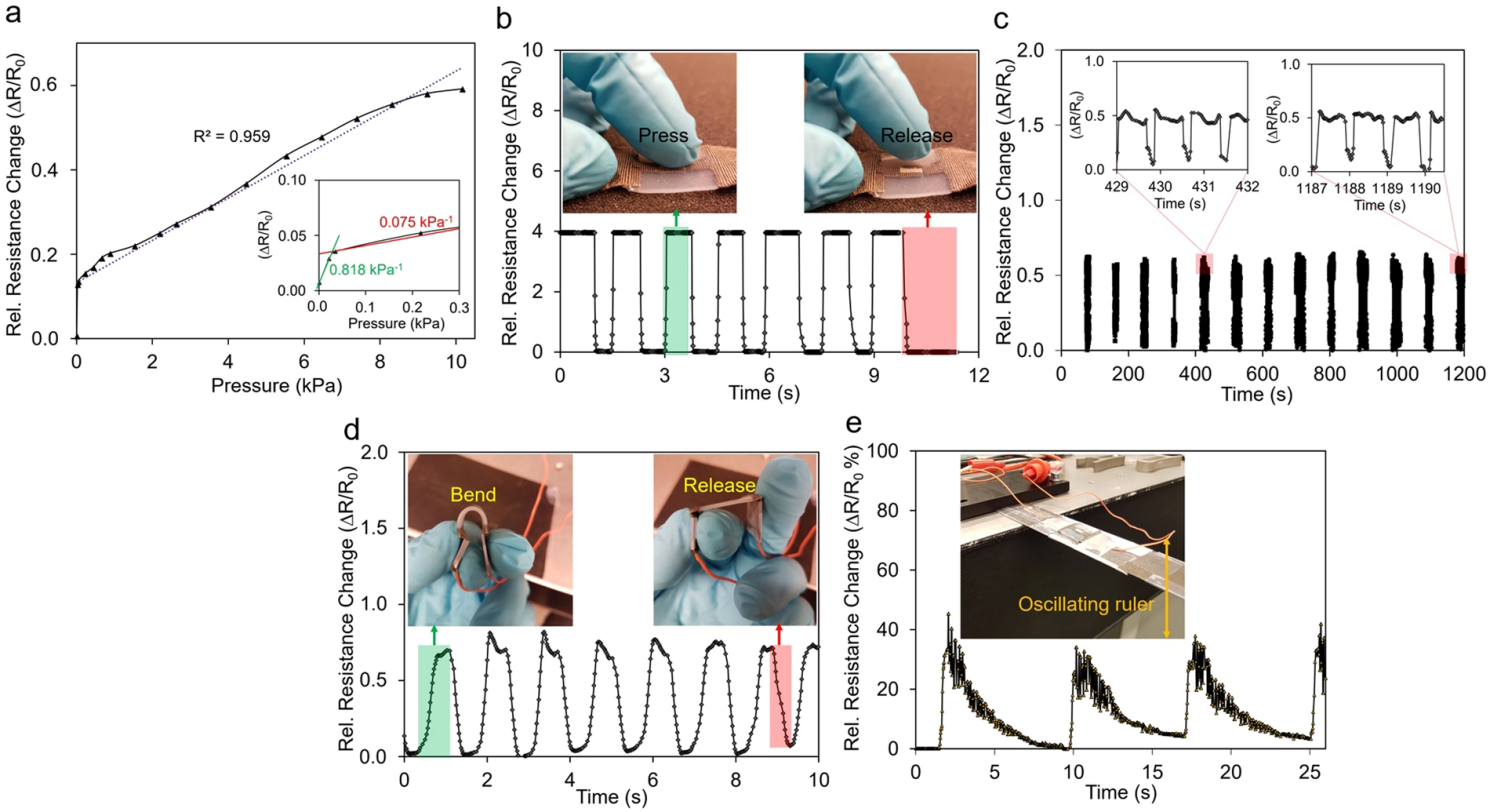
Measurement results for pressure, bending, and vibration. The relative change to the applied pressure of 0–10 kPa for Ag-DS/CF when it is distributed evenly over the area of DS without the pre-stress (**a**). The dynamic response of Ag-DS/CF to applied pressure of finger touch (**b**). The relative resistance change versus applied pressure of 10 kPa for over 1000 repeated cycles with the pre-stress. The response was measured after each 100 repeated cycles (**c**). The Ag-DS/CF response to bending between fingers to approximately bending angle of 180 degrees (**d**). The time-dependent response of Ag-DS/CF to oscillating steel ruler (**e**). (R_0_ = 300 Ω).

Figure 4a shows the response of an Ag-DS/CF to an applied pressure of 0–5 kPa, where the pressure was distributed evenly over the area over-molded with DS. The relative resistance change increased relative linearly in three pressure regimes of 0–0.035 kPa, 0.035–0.200 kPa, and 0.200–10 kPa showing maximum pressure sensitivity of 0.82 kPa^−1^ decreasing to 0.075 kPa^−1^ and 0.051 kPa^−1^ as the pressure further increased. The pressure sensitivity of Ag-DS/CF is related to the relatively high GF (>130) obtained at small strains (<1%) and the extreme softness of the used elastomer, as the applied pressure induced only a small strain to the Ag-DS/CF in this pressure regime.

The pressure sensitivity was further studied by observing the dynamic response of Ag-DS/CF to a dynamic press-release motion from a light finger touch directly above the cavity at a frequency of approximately 0.8 Hz (Fig. 4b). The Ag-DS/CF shows a highly reproducible and extremely stable response to the applied pressure that was independent of the frequency of the mechanical stimuli. The relative resistance change was more profound as the cavity was pressed, instead of distributing the applied pressure on the total area covered by the DS. As a result, an extremely low detection limit of <1 Pa for pressure was observed in Ag-DS/CF by placing a small piece of paper (36 mg) on the surface of the sensor above the cavity. The obtained detection limit is comparable to that found in other strain sensors with graphite thin films, carbonized silk, and coated PVDF yarns as the active materials (Table S1). Similar pressure sensitivities at low pressure ranges (<10 kpa) were achieved in other studies. However, much higher pressure sensitivities than in Ag-DS/CF were demonstrated with the use of PANI, coated PVDF yarns, and graphene oxide (18.4–37.6 kPa^−1^) as the active materials (Table S1) but achievable GF (<200) were much lower than reported in this work. The cavity-design of Ag-DS/CF could be further utilized in fabricating even more sensitive pressure sensors but that would be expected to have a negative effect on the strain sensing properties.

The response and recovery times of Ag-DS/CF were observed to be approximately 70 and 200 ms for press-release cycles by finger touch (Fig. S8). The longer recovery times observed could be related to the hysteresis of the Ag-DS/CF increasing the settling time of the resistance value after release of the pressure. However, the response and recovery times were highly dependent on the type of mechanical stimuli (e.g. pressure, strain) and the speed of the stimuli and thus would be expected to noticeably decrease at higher frequencies for pressure sensing.

As shown in Fig. 4c, the response of Ag-DS/CF exhibited a high stability during >1000 repeated cycles at 10 kPa pressures with a frequency of 1.3 Hz and the pre-stress of approx. 1 kPa when using the computer controllable piston, Festo. The responses of the sensor were measured after each 100 repeated cycles for approximately 20–50 cycles. The dynamic performance during durability testing showed reproducibility and stability indicating no degradation of the properties over the multiple cycle test. The small non-uniformity of the signal shape is associated with the testing setup, as the sample could slightly twist during the press-release cycles^51^ when perpendicular attachment to press-direction and pre-stressing of the sample was required^31^, and because the movement of the Festo showed time-dependent variation.

### Dynamic bending and sensing of vibration

The relative resistance changes for dynamic bending to an angle of approximately 180 degrees of Ag-DS/CF between the fingers at a frequency of approximately 0.85 Hz was recorded (Fig. 4d). The strain sensors showed reproducibility and stability to bending with relative resistance changes of approximately 67–80%. The variation in the response of the sensor could be related to differences in the bending during dynamic testing. The overshooting and undershooting could be associated with the complexity and direction of the applied stress in respect to that of the pre-stretching direction during the fabrication process. Regardless, the Ag-DS/CF could be expected to be able to detect the change in the bending angle and motion reliably, and that would be advantageous in human/machine interfaces, robotics, etc.

In addition to bending, detection of physical vibrations was demonstrated when Ag-DS/CF were attached to an oscillating steel ruler (Fig. 4e). The strain sensors showed a repeatable signal pattern during mechanical vibrations, where even the smallest vibrations of the damped oscillations of the ruler could easily be detected. For mechanical vibrations, a fast response time of approximately 20 ms was achieved with Ag-DS/CF (Fig. S9); faster than in most reported strain sensors (Table S1) and comparable to that found in some cases^34^. The possibility to sense physical vibrations enables versatile applications related to other areas, such as detection of the condition of structures and machine maintenance.

### Human motion monitoring

**Figure 5:**
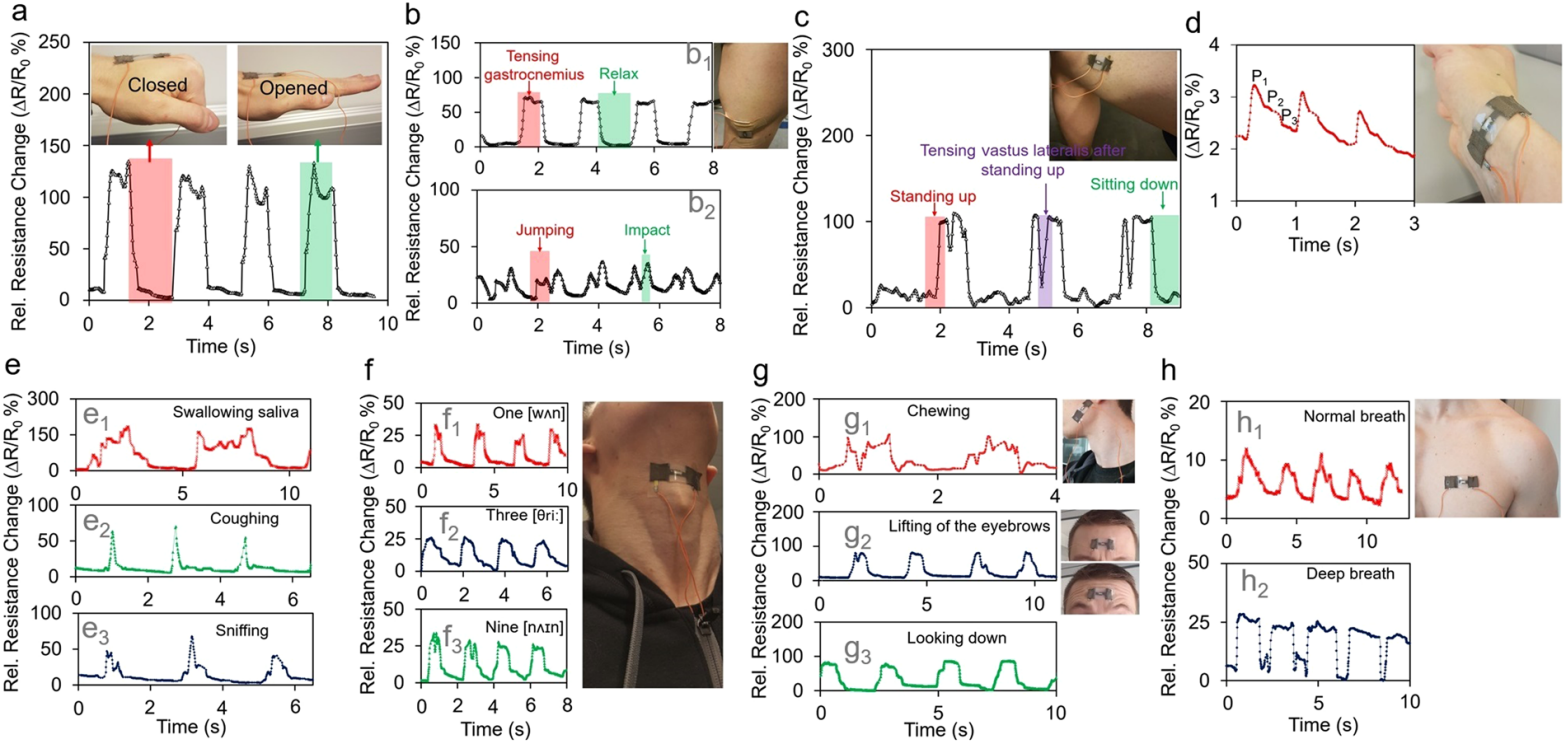
Measurements results for subtle signals and large scale motions associated with the human body. Photographs showing the Ag-DS/CF strain sensor fixed onto the back of the hand (**a**), gastrocnemius muscle (**b**), vastus lateralis (**c**), wrist (**d**), neck (**e**,**f**) cheek (g_1_), forehead (g_2_), and lower neck (g_3_), and chest (**h**). The corresponding time dependent signals of opening and closing fist (**a**), muscle tension of gastrocnemius muscle when doing one legged seating calf raises (b_1_) and jumping with two feet (b_2_), muscle tension of vastus lateralis when standing up from seated position and tensing the muscle after standing up (**c**), wrist pulse (**d**), swallowing of saliva (e_1_), coughing (e_2_), sniffing (e_3_), and phonation of words one (f_1_), three (f_2_), and nine (f_3_), chewing (g_1_), motion of forehead when lifting the eyebrows (g_2_), and head movement when looking down (g_3_), and chest movement during normal (h_1_) and deep breathing (h_2_). (R_0_ = 300 Ω).

Figure 5a shows the response of an Ag-DS/CF when attached to the back of the hand with the re-usable adhesive layer during repeated fist opening-closing cycles. The response showed a repeatable pattern, with high overshoot and undershoot, somewhat similar to the response to bending (Fig. 4d), that could be partly related to the complexity of the nature of the strain, as the movements of the tendons are multidirectional. Also, sliding of the strain sensor was extremely likely during the continuous measurement and this would induce both overshooting and undershooting behavior of the strain response. A multistimuli was applied by using fist opening-closing cycles while simultaneously touching the surface of the sensor with a finger once within each cycle (Fig. S10). The multistimuli can be distinct from the single stimulus (i.e. seen in Fig. 5a) as higher response and two-peaked signal shape (Fig. S10) with a single transduction mechanism due to the ultrasensitivity and fast response time of the sensor.

For more complex motions associated with the human body, the Ag-DS/CF were attached with flexible tape to the gastrocnemius muscle (Fig. 5b) during tensing of the muscle by doing one-legged seated calf raises (Fig. 5b_1_), and when moderately jumping on the spot with two feet (Fig. 5b_2_). The Ag-DS/CF showed a highly repeatable and constant signal with relative resistance changes of approximately 65% during the one-legged seated calf raises with slight undershoot and overshoot. For the jumping manoeuvre, the Ag-DS/CF showed a repeatable signal response, where the first and the second peaks corresponded to the take-off where the calf tenses for short period of time, and to landing, where the calf tenses again to dampen the impact. Periods of approximately 500 ms between the initial jumping motion and the impact when landing (Fig. 5b_2_) were observed.

For an investigation of other complex motions of the human body, the Ag-DS/CF were attached to the vastus lateralis (Fig. 5c, side of the thigh), to monitor the combined motion of standing up from the seated position, and subsequently to monitor the tensing of the vastus lateralis before sitting down again. The Ag-DS/CF showed a repeatable signal with a two-peaked response, similar to that of the jumping motion. The standing up motion increases the tension of the vastus lateralis (the first peak), whereas the muscles eventually relax when standing still, before the muscles are purposely tensed again (the second peak) before finally sitting down.

In addition, it was observed that the response to tensing the vastus lateralis was higher than that of the gastrocnemius. That could be related to the size of the muscle inducing a higher strain during motion (Fig. S11). As seen in Fig. S11, when the Ag-DS/CF were attached to forearm, bicep, side of chest, and tricep to monitor the muscle tension of flexor carpi (Fig. S11a), pectoralis major (Fig. S11b), bicep (Fig. S11c), and lateral heads of triceps brachii (Fig. S11d), when performing one-arm wrist curl, bottom half motion of bicep curl, end motions of peck fly and tricep extension without weights, respectively, the size of the muscle correlated with the response of the strain sensor. The Ag-DS/CF showed no frequency dependency at ≤1 Hz during motion (Fig. S11a–c) and were highly stable under longer periods of holding muscle tension (Fig. S11d). The response could be affected by individual factors such as body fat and shape of the muscle, because the lower the body fat and the fuller the muscle bellies are the more uneven the surfaces of the skin when the muscles are tensed. Also, an individual’s trained capability to tense the muscle harder during movement leads to an increased response of the Ag-DS/CF when attached to specific location on the body. Due to their extraordinary tracking ability during complex motions of the human body, and their ability to record the degree of muscle tension, the Ag-DS/CF show great potential for virtual reality and interactive gaming applications, for evaluating and tracking of sports performance through movement analysis, and in recording the effectiveness of individual training during weight lifting.

The wrist pulse provides a lot useful and valuable information for non-invasive medical diagnosis. Thus, to demonstrate the Ag-DS/CF strain sensors capability to detect arterial blood pressure and their potential to act as wearable diagnostic devices, a sensor was tightly attached, using the fabricated and re-usable adhesive layer, to the wrist of a healthy 28-year old male with height of 180 cm and weight of 78 kg (Fig. 5d). The wrist pulse was measured after the subject had been resting for several minutes in the seated position. The Ag-DS/CF showed a clear response with a regular and repeatable pulse shape providing detailed information about the individual’s state of health. The measured pulse waveform clearly revealed the typical characteristic peaks associated with percussion wave (P_1_), tidal wave (P_2_), and diastolic wave (P_3_). The central arterial stiffness (AI), expressed as AI = (P_1_ − P_2_)/PP, where PP is the absolute pulse wave amplitude, and pulse wave velocity (PWV), expressed as RI = h/ΔT, where h is height of the subject and ΔT is the time interval between P1 and P3, were calculated to be 27.47% and 3.78 ms^−1^, respectively, that are in line with normal values for a healthy individual. The central AI and PWV values can be used as markers to detect early vascular damage and cardiovascular disease risk. Furthermore, the signal showed a heart rate of approximately 63 beats min^−1^ that corresponded with simultaneous optical wrist pulse measurement with a Polar A360 on the other wrist. However, time-dependent drifting of the signal for the wrist pulse measurement was observed, that could be related to signal variation due to the small relative resistance change due to blood pressure variation (approx. 1.5%) and heating of the sensor caused by generated body heat during the long testing period, as the Ag-DS/CF were observed to be temperature-sensitive.

In addition to wrist pulse measurements, the strain sensor was attached to the neck above the Adam’s apple to monitor subtle physiological motions of the thyroid cartilage bone and vocal chords due to saliva swallowing (Fig. 5e_1_), coughing (Fig. 5e_2_), sniffing (Fig. 5e_3_), and phonation of words (Fig. 5f_1–3_), respectively. The time-dependent response to saliva swallowing showed a high relative resistance change of approximately 170% with one or two peaks depending whether the swallowing was one at once, or whether the initial swallowing was followed by multiple smaller swallowing motions. For coughing, relative resistance changes of approximately 36–65% were observed with a non-reproductible signal shape. For saliva swallowing and coughing, the non-reproducibility of the signal was observed to be partly associated with variations in muscle movements in the subject’s neck each time saliva was swallowed or when the subject coughed. For sniffing the acquired signal was highly reproducible and repeatable with relative resistance change varying between 50–65%. However, no signal drift during measurements was observed. Figure 5f shows the detection of phonation when the numbers “One”, “Three”, and “Nine” were pronounced. The signal patterns between words were highly distinguishable from each other, resulting from the particular motions of the vocal chords, and were reproducible, confirming the reliability of the Ag-DS/CF to detect speech. The Ag-DS/CF response was observed to be associated with the volume of pronunciation. Thus, the strain sensor shows outstanding potential for the monitoring of an individual’s state of health for rehabilitation exercises and for human/machine interactions where the detection of speech is required.

Monitoring of eating patterns, especially in elderly people, has great importance for human health as sufficient nutrient intake is required to prevent many health problems associated with malnutrition. To demonstrate an Ag-DS/CF ability in the monitoring of eating patterns, it was attached to the cheek of the subject to monitor the chewing motion during eating (Fig. 5g_1_). The time-dependent response of the Ag-DS/CF showed a high relative resistance change of approximately 100%, while the signal pattern varied with time due to differences in the degree of muscle movements when eating a real meal. Improved symmetry of signal pattern could be achieved with a new adhesive layer over-mold on top of the old one or by using medical tape to improve the attachment, as underestimation of the strain had previously been observed that could indicate sliding of the sensor over the skin^35^. However, the signal pattern was clearly distinct from other motions (e.g. saliva swallowing), indicating that periods of eating can be reliably detected with Ag-DS/CF.

In addition, detection of facial expression and movement of the head have great importance e.g. in human/machine interfaces for robotics, and in monitoring the state of mind through emotions. Thus, Ag-DS/CF were attached with the re-usable adhesive layer to the muscles around the forehead frontalis (Fig. 5g_2_) and lower neck (Fig. 5g_3_) to detect lifting of the eyebrows and movement of the head when looking down, respectively. The Ag-DS/CF showed a clear response with relatively repeatable signal patterns in both cases, whereas differences in the degree of muscle movements of the forehead frontalis slightly altered the response of the Ag-DS/CF.

Also, another important physiological signal for human health is the respiration rate that can increase with illness and other medical conditions. To demonstrate the potential application of Ag-DS/CF as a respiration rate monitor, it was attached to the chest of healthy 28-year old male with the re-usable adhesive layer (Fig. 5h). The respiration rate was monitored when the subject was in a relaxed seated position. The Ag-DS/CF produced repeatable and regular signal patterns, where upward and downward slopes of the response correlated with chest expansion during inhalation and chest contraction during exhalation, respectively. The respiration rates for normal breathing were observed to be 20 breaths min^−1^; these were in line with normal respiration rates of 12–20 breaths min^−1^ for an adult during relaxation. A higher respiration rate of 30 breaths min^−1^ and the shape of the signal was associated with purposely breathing faster and holding the breath for a short period of time before exhaling to test the strain sensor’s response and capability to detect abnormality in respiration rate.

The extreme conformability and outstanding performance, related to the high sensitivity and wide working range and versatility of the Ag-DS/CF strain sensors allowed their use as skin-attachable wearable devices for real-time monitoring of large scale and complex motions, and also subtle physiological signals associated with the human body. The sensor was able to reliably detect jumping, sitting-standing motion, muscle tensions and motions at different locations of the body, movements of the Adam’s apple (e.g. coughing, phonation of words), chewing motion when attached to cheek, wrist pulse and respiration rate due to blood pressure and chest movement, respectively. Remote and personalized health-care is a promising route to improve diagnosis and early treatment of diseases. The need for sensors in virtual reality based interactive gaming, rehabilitation, tracking sports performance, and fitness is increasing. For all these applications the Ag-DS/CF show remarkable potential.

## Conclusion

In summary, extremely versatile and machine washable strain sensors with high stretchability, ultrasensitivity, and tunable sensing ranges have been developed. The strain sensors were based on a unique structure combining pre-stretching induced buckling and cavity design to create a wrinkling effect in an Ag-ink pattern, and uniquely shaped silver-plated nylon fabrics in silicone elastomer. The strain sensors exhibited extremely high GFs of >10^4^–10^6^ with tunable sensing ranges from 7 to 75%, low hysteresis of <20%, low overshoot of 2.5%, and high durability of >3000 repeated cycles at 20% strain. A maximum pressure sensitivity of 0.82 kPa^−1^ at 0.035 kPa was achieved with a minimum detection limit of <1 Pa. Also, a high durability of >1000 cycles with a pressure of 10 kPa was obtained. The sensors had minimum response times to physical vibrations of approximately 20 ms. The achieved properties enabled the sensors to detect a wide range of signals from large-scale motions (e.g. muscle movements) to subtle deformations (e.g. wrist pulse, phonation of words), pressure, and physical vibrations. The designed strain sensor provides a promising platform for the future of wearable sensors for the versatile monitoring of human health and motion.

## Methods

### Materials

The Smooth-On Inc Dragon Skin Fast 10 silicone rubber and Skin Tite silicone were purchased from Silcom Oy (Finland). The MedTex P130 silver-plated nylon was supplied by Kitronik (United Kingdoms). The conductive silver ink CI-1036 was provided by Global Print Solutions Limited (United Kingdoms).

### Fabricating structure for printing

Large piece of CF was cut into two T/L-shaped CFs (Fig. S1a) that were secured by double-side tape onto bottom of a mold. Multiple pieces of KTs were stacked onto each other. The KT was cut into rectangular shape with dimensions of 5 mm × 1 mm × 0.2 mm, that was placed between the L-shaped elongations of CFs (Fig. S1b). The A- and B-components of DS were combined in a ratio of 50:50, mixed thoroughly and then poured into the mold over the DS/CF-structure. The DS/CF-structure was left to dry for over 75 minutes at room temperature before removing the DS/CF-structure from the mold and KT. The KT ultimately formed a cavity to the inverted DS/CF-structure.

### Fabrication of ink pattern

The silver ink was screen-printed into the cavity of the inverted DS/CF-structure. The inverted and pre-stretched DS/CF-structure was cured at 120 °C for 10 minutes. After curing, the pre-stretching was released to generate a wrinkled Ag-pattern. The Ag-DS/CF was over-molded with another layer of DS to cover the cavity and the sample was left to dry for several hours at room temperature.

### Fabrication of adhesive layer

The A- and B-components of the Skin Tite with a ratio of 50:50 were mixed and spread thinly onto the backside of the Ag-DS/CF. The sample was left to dry for >60 minutes at room temperature.

### Characterization

The electrical properties of Ag-DS/CF-strain sensors were measured with a Keysight Electrometer B2987A at room temperature. The samples were fixed to a stretching and/or bending tester consisting of an actuating unit and a stationary part. The long-term stability of the sensor for dynamic pressure testing was achieved with a computer controllable piston (Festo) when using a pre-stress. Static pressures were applied by using weighted objects on platform. Mechanical properties were measured with LINKAM TST350 Tensile Stress Testing system with 200 N force transducer.

### Ethical consent

According to the Finnish Advisory Board on Research Integrity (TENK), this study is not subject to ethical review.

## Electronic supplementary material

Supplementary information

## Data Availability

The data that support the findings of this study are available from the corresponding author upon reasonable request.
