# Supplementary material for: Stretchable and Washable Strain Sensor Based on Cracking Structure for Human Motion Monitoring
Source: Sci Rep. 2018 Sep 5;8:13241. doi: 10.1038/s41598-018-31628-7 (PMC6125599; doi:10.1038/s41598-018-31628-7)
Supplement: Supplementary file 1 — Supplementary information [file 41598_2018_31628_MOESM1_ESM.pdf]

Supporting Information

Stretchable and Washable Strain Sensor Based on Cracking Structure for Human Motion Monitoring

Jarkko Tolvanen, Jari Hannu, and Heli Jantunen*

Microelectronics Research Unit, Faculty of Information Technology and Electrical Engineering, University of Oulu, Oulu 90014, Finland

E-mail: jarkko.tolvanen@oulu.fi

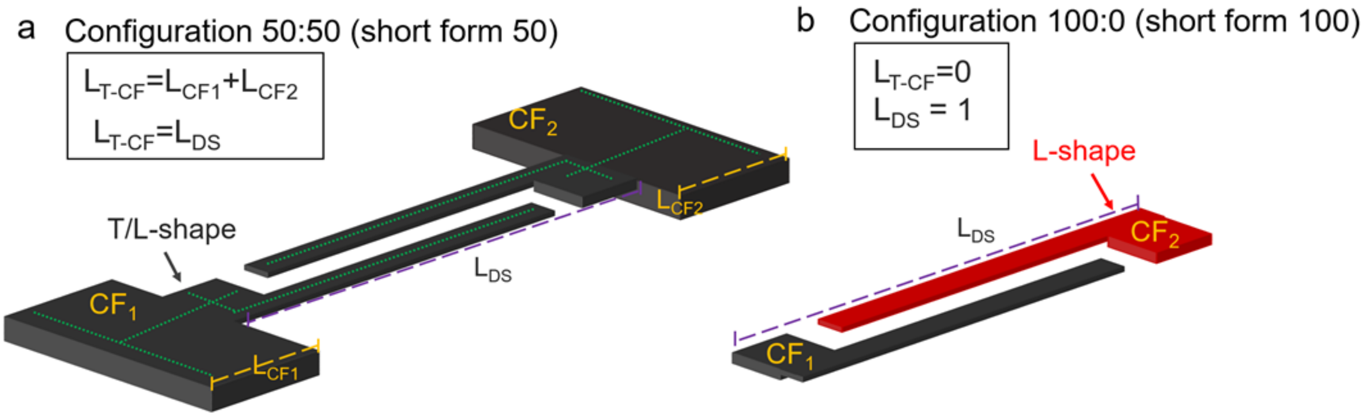

Figure S1. The structural differences between sensor configurations 50:50 and 100:0. As the configuration transforms from 50:50 (**a**) to 100:0 (**b**), the shape of the CFs changes from T/L-shape to L-shape. The L_{T-CF} is the total length of the CFs outside the DS.

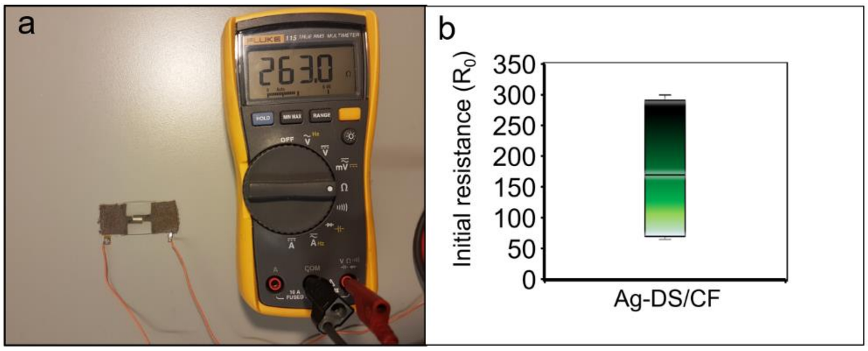

Figure S2. Initial resistances of the strain sensors. The photograph of the Ag-DS/CF showing initial resistance of 263 Ω when measured with a multimeter and no stretching is applied (a). The variation of the initial resistances between > 10 fabricated samples shows median value of 170 Ω with upper and lower boundaries of 300 Ω and 65 Ω, respectively.

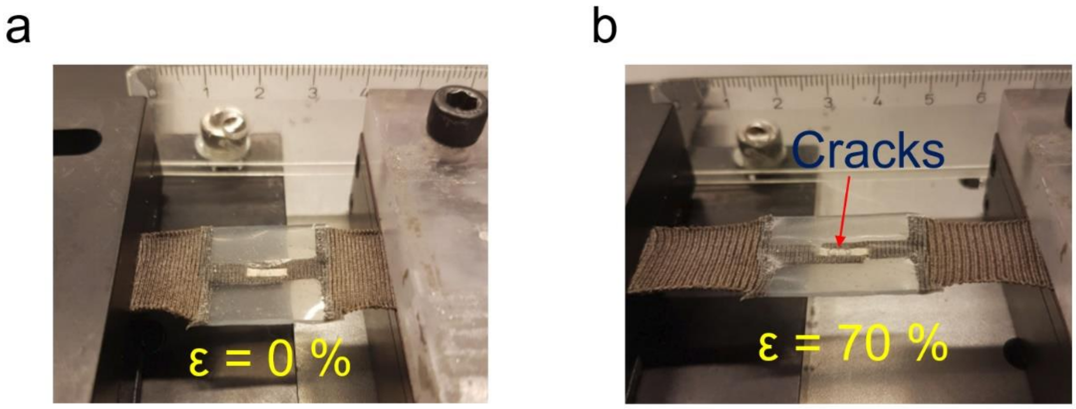

Figure S3. Stretching strain sensor with the tester. Stretching of Ag-DS/CF strain sensor from 0 (**a**) to 70% (**b**) with stretching and/or bending tester. The cracks form perpendicular to direction of stretching (**b**). The electrical contact from the Ag-DS/CF to the tester was made by placing two brass conductors with soldered wires to both bottom clamps on both sides of the tester. The ends (CF) of the sensor were pressed against the conductors with top clamps.

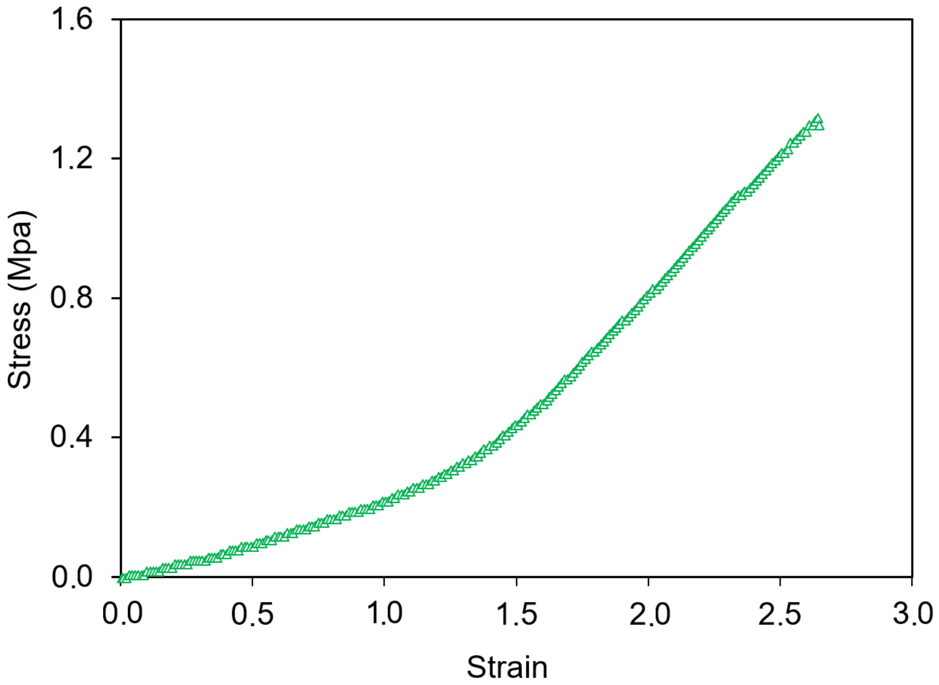

Figure S4. Stress-strain curve. The recorded stress-strain curve with tensile stress testing system for Ag-DS/CF with sensor configuration 80.

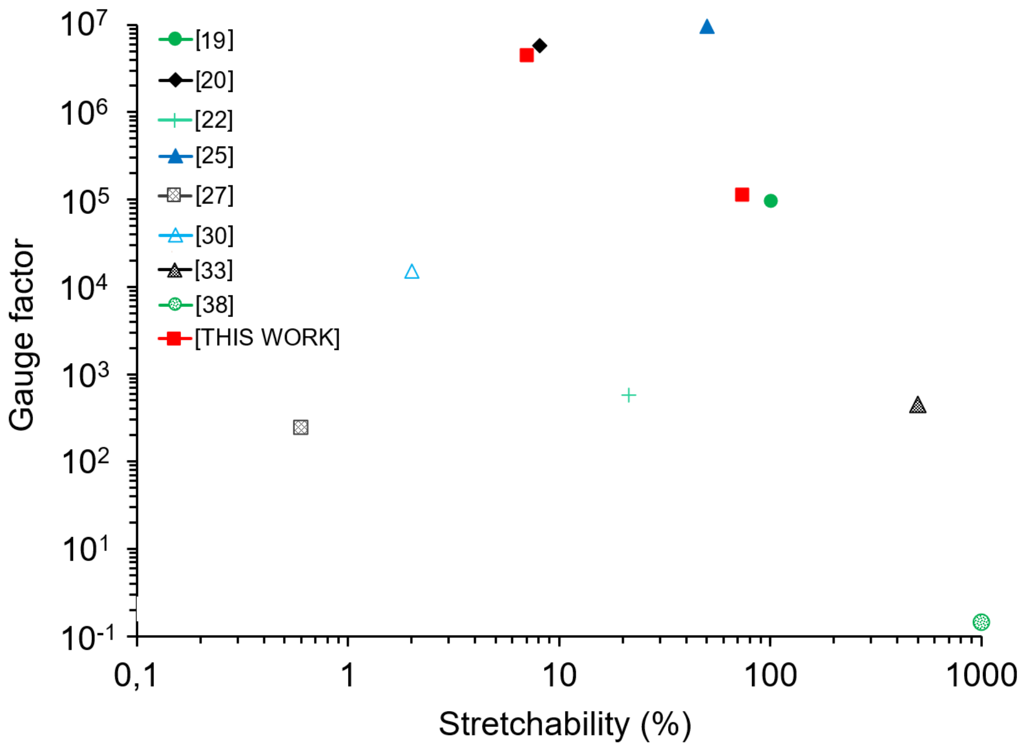

Figure S5. Comparison of advanced strain sensors' sensitivity (gauge factor) to stretchability. The gauge factors shown in this figure are measured at the indicated strain.

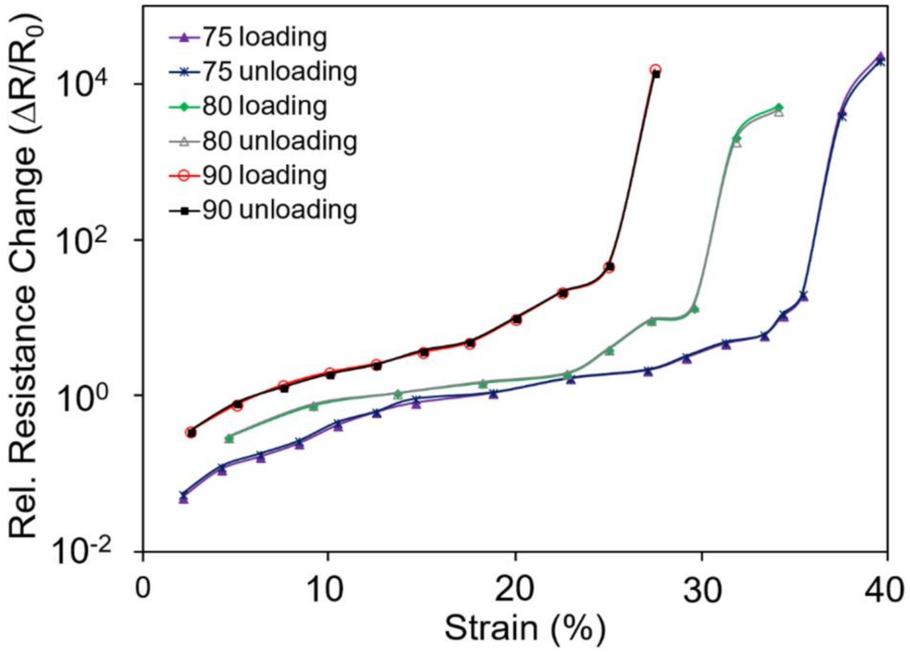

Figure S6. Hysteresis curve. The recorded hysteresis for Ag-DS/CF 75, 80, and 90 sensor configurations during loading-unloading cycles. (R_0 = 300 Ω)

Table S1. Comparison of Ag-DS/CF strain sensors' properties to others found in the literature.

Active Material	GF [a)]	ST [b)] [%]	DB [c)]	OS [d)] [%]	HYS [e)] [%]	S [f)] [kpa-1]	Range [kPa]	RT [g)] [ms]	DT [h)]	MP [i)]	MS [j)]	Ref
PVDF yarns coated with PEDOT	*	*	7.5k (10 kPa)	-	-	18.4	10	15	15 mg	-	SP	[3]
GO doped PU/PEDOT	193	550	10k (120%)	-	-	20.6	20	-	0.5%	-	SP	[4]
AgNW	20	35	-	-	-	-	-	-	-	Y	-	[7]
PANI	11.6	100	15k	-	-	37.6	> 5	50	-	-	SP	[10]
AgNW	150k	60	-	-	-	-	-	-	-	-	-	[11]
MWCNT	1378	600	10k (100%)	-	-	-	-	-	-	-	-	[13]
$Ti_3C_2T_x$ MXene/CNT	772	70	5k (20%)	-	-	-	-	-	0.1%	-	-	[17]
AgNW/POE	13.9K	64	4.5k (10%)	-	-	-	-	<10	0.065	-	-	[18]
PEDOT:PSS/SWCNT	100k	100	1k (20%)	-	-	-	-	<72	-	-	-	[19]
SWCNT paper	10M	50	10k (20%)	-	-	-	-	300	-	-	-	[20]
SWCNT thin film	*	*	68k (1kPa)	-	-	1.8	1.2	-	-	-	SP	[21]
RGO microtubes	630	43	400 (14%)	-	-	-	-	-	-	-	-	[22]
Graphene film	1037	4.4	-	-	-	-	-	-	-	-	-	[23]
Graphene woven fabric	6M	10	1k (3%)	-	-	-	-	< 72	-	-	-	[25]
DMSO-doped PEDOT: PSS	280	1	2k	-	-	-	-	-	<0.2	-	-	[27]
Crystalline silicon nanoribbon	12	30	-	-	-	-	200	-	0.4%	-	SPTH	[29]
Pt	16k	2	-	-	-	-	-	-	-	-	-	[30]
Graphene/AgNW	476	500	1k (50%)	-	-	-	-	-	0.5%	-	-	[33]
Carbonized silk	173	100	10k (10%)	-	-	0.19	10	70	0.01%	-	SP	[34]
RGO woven fabric	3670	57	1k (7.5%)	-	-	-	-	20	-	-	-	[39]
Ag ink/Ag plated nylon	4.65M	75	3k (20%)	2.5	3	0.82	10	20	1 Pa	-	SP	[This work]

[a)]gauge factor (maximum value); [b)]stretchability in percentage (maximum value), [c)]durability; [d)]overshoot in percentage (lowest value); [e)]hysteresis in percentage (lowest value); [f)]sensitivity for pressure (maximum value); [g)]response time (minimum value); [h)]detection limit for strain/pressure; [i)]multiplane sensing in xy-direction; [j)]multisensing (S=strain, P=pressure, T=temperature, H=humidity) (* = was applied for human motions with low strains (i.e. wrist pulse))

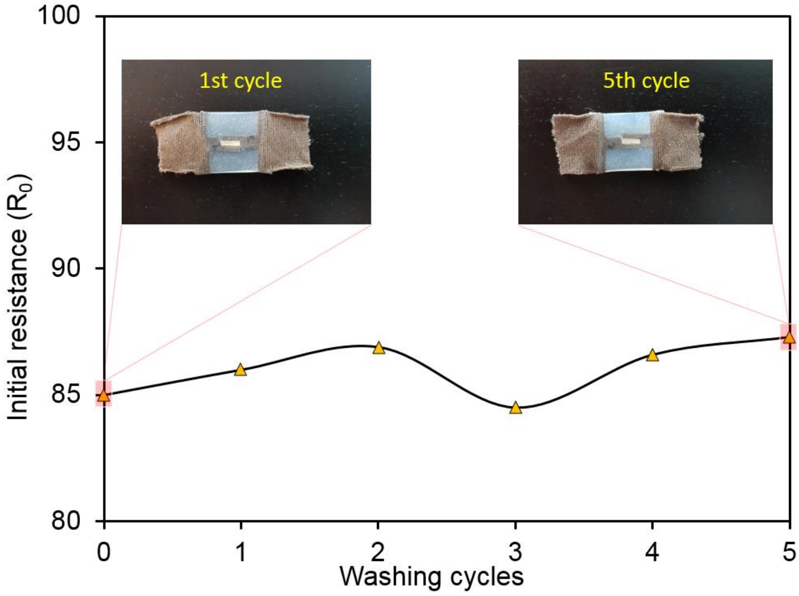

Figure S7. Initial resistance change between machine washing cycles. The initial resistance change of Ag-DS/CF strain sensor during repeated washing cycles at temperature of 40℃ with 1200 rpm for 30 minutes within each cycle. The structure stayed intact and showed no mechanical failure associated with the increase of the initial resistance value. ($R_0 = 85\ \Omega$)

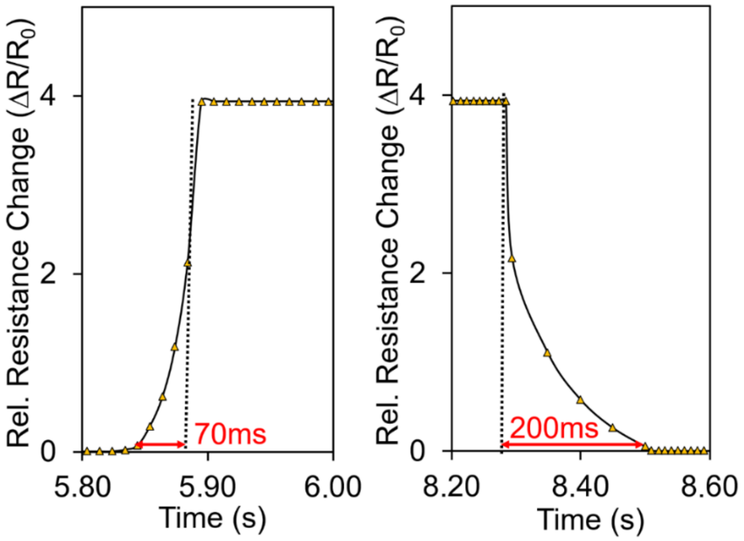

Figure S8. Response and recovery times. The response and recovery times of Ag-DS/CF when touching with a finger at frequency of 0.8 Hz.

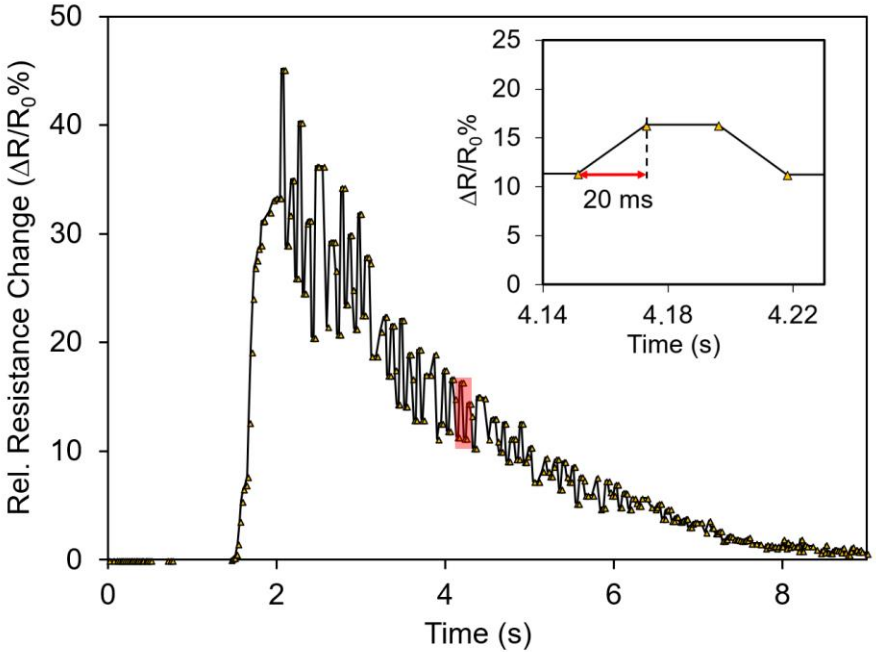

Figure S9. Response time to physical vibrations. Time dependent response of Ag-DS/CF to oscillating steel ruler revealing the response time of the sensor. ($R_0 = 284\ \Omega$)

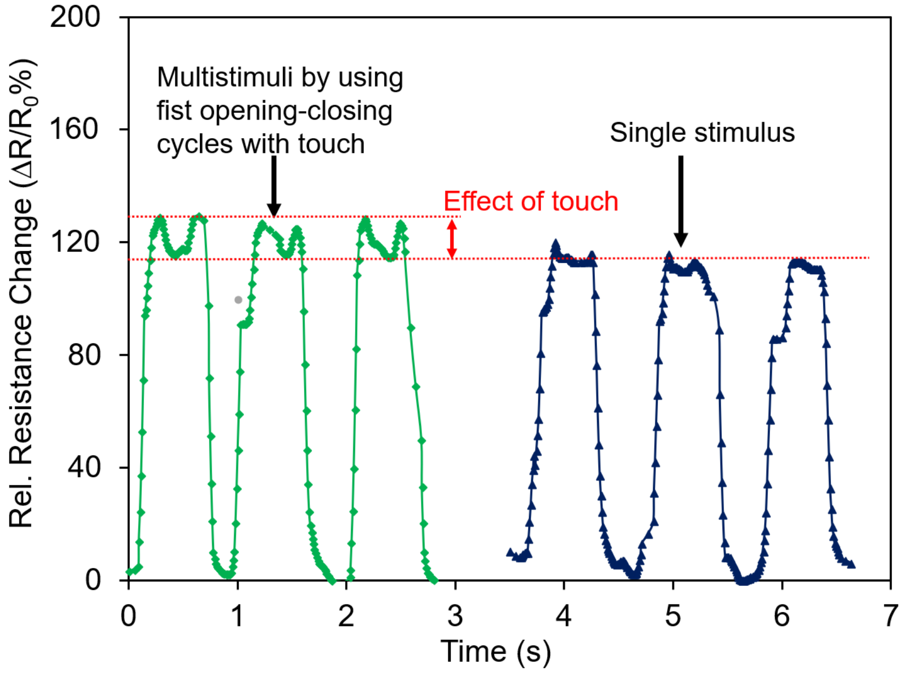

Figure S10. Response to applied multistimuli. The sensor was attached to back of the hand. Simultaneous fist opening-closing cycles with a finger touch were applied, that were compared to single stimulus with fist opening-closing cycles. Two tests were plotted on a single graph for comparison. ($R_0 = 284\ \Omega$)

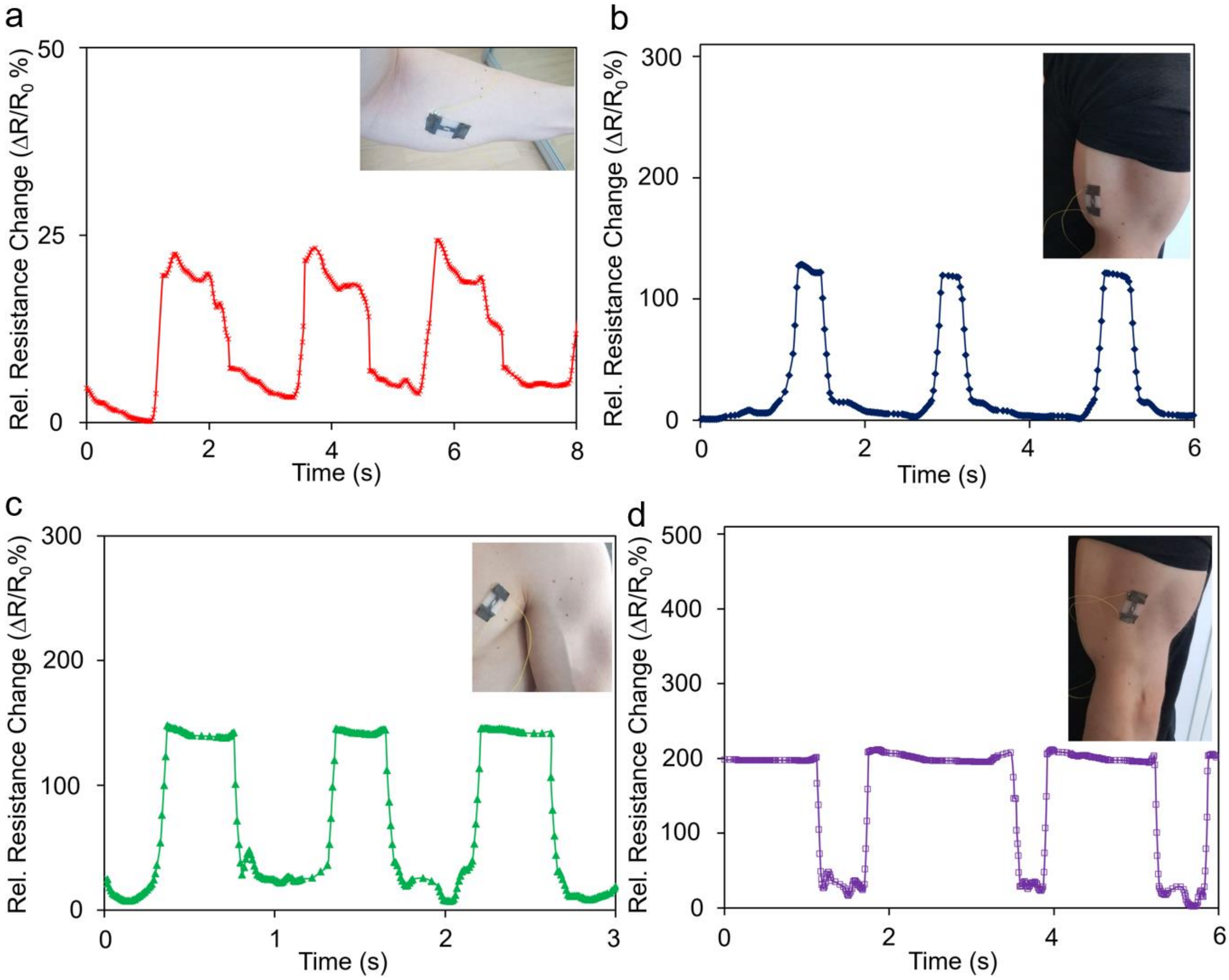

Figure S11. Measurement results for muscle tensions. Photographs showing the Ag-DS/CF strain sensor fixed onto forearm (**a**), bicep (**b**), side of chest (**c**), and tricep (**d**). The corresponding time dependent signals of muscle tension of flexor carpi (a), pectoralis major (**b**), bicep (**c**), and lateral head of triceps brachii (**d**). ($R_0 = 300\ \Omega$)
